# *Inonotus sanghuang* Polyphenols Attenuate Inflammatory Response Via Modulating the Crosstalk Between Macrophages and Adipocytes

**DOI:** 10.3389/fimmu.2019.00286

**Published:** 2019-02-26

**Authors:** Mengdi Zhang, Yu Xie, Xing Su, Kun Liu, Yijie Zhang, Wuyan Pang, Junpeng Wang

**Affiliations:** ^1^Institute of Infection and Immunity of Huaihe Hospital, Henan University, Kaifeng, China; ^2^School of Physical Education, Henan University, Kaifeng, China; ^3^College of Biology Science and Engineering, Hebei University of Economics and Business, Shijiazhuang, Hebei, China

**Keywords:** *Inonotus sanghuang*, polyphenols, inflammation, obesity, NF-κB signaling, MAPK signaling

## Abstract

**Aims:** Obesity is characterized as a chronic state of low-grade inflammation with progressive immune cell infiltration into adipose tissue. Adipose tissue macrophages play a critical role in the establishment of chronic inflammatory states and metabolic dysfunctions. *Inonotus* (*I*.) *sanghuang* and *its* extract polyphenols exhibit anti-carcinogenesis, anti-inflammatory, and anti-oxidant activities. However, the action of *I. sanghuang* polyphenols in obesity-related inflammation has not been reported. The aim of this study was to explore the anti-inflammatory action of polyphenols from *I. sanghuang* extract (ISE) in macrophages and the interaction between macrophages and adipocytes.

**Materials and Methods:** RAW264.7 macrophages were stimulated with LPS or conditioned medium of hypertrophied 3T3-L1 adipocytes or cocultured with differentiated adipocytes in the presence of different doses of ISE. The inflammatory cytokines were evaluated by ELISA, the MAPK, NF-κB, and IL-6/STAT3 signals were determined by immunoblotting, and the migrated function of macrophages was determined by migration assay.

**Results:** ISE suppressed the inflammatory mediators including NO, TNF-α, IL-6, and MCP-1 induced by either LPS or conditioned medium derived from 3T3-L1 adipocytes. ISE also decreased the production of these inflammatory mediators in cocultures of 3T3-L1 adipocytes and RAW264.7 macrophages. Furthermore, ISE blocked RAW264.7 macrophages migration toward 3T3-L1 adipocytes in cocultures. Finally, this effect of ISE might be mediated via inhibiting ERK, p38, and STAT3 activation.

**Conclusions:** Our findings indicate the possibility that ISE suppresses the interaction between macrophages and adipocytes, attenuates chronic inflammation in adipose tissue and improves obesity-related insulin resistance and complication, suggesting that ISE might be a valuable medicinal food effective in improving insulin resistance and metabolic syndrome.

## Introduction

Obesity is a medical condition in which excess body fat has accumulated to the extent that it may have a negative effect on health. Obesity prevalence has doubled since 1980 and been continuously increasing. In 2015, high BMI accounted for 4.0 million deaths globally, nearly 40% of which occurred among non-obese people (1).

Obesity is a chronic disease, and studies (2) have shown that obesity is not only related to the occurrence of a variety of chronic diseases, but also a risk factor for these chronic non-communicable diseases such as type 2 diabetes mellitus (T2DM), hypertension, coronary heart disease, and strokes (3). Not only does obesity affect health, but also the complications it causes have become some of the main disease burdens worldwide. However, the metabolic response to obesity is diverse; a growing amount of evidence suggests that there is a considerable proportion of obese individuals that lack obesity-associated diseases and are metabolically healthy (4). Inflammation is one of the causes that distinguish metabolically healthy from metabolically unhealthy obesity. Obesity-related inflammation is mainly caused by cytokines secreted from adipose tissue macrophages (ATMs) (5). Inflammation that ATMs drive links obesity to insulin resistance, which is a central mechanism in obesity-associated diseases such as T2DM and metabolic syndrome (6). With progressive obesity, ATMs are crucial mediators of meta-inflammation, insulin resistance, metabolic dysfunction, and have other bad influences on adipocyte function (7).

ATMs are distributed between adipocytes and along vascular structures in adipose tissue, and they secrete anti-inflammatory mediators such as IL-10 and catecholamine which regulate adipocyte lipid metabolism. Resident macrophages in tissue show prodigious heterogeneity in their activities and functions, primarily reflecting their local metabolic and immune microenvironment (8). There is substantial evidence that ATMs exhibit the phenotypic change from M2 or “alternatively activated” (anti-inflammatory) macrophages to M1 or “classically activated” (pro-inflammatory) macrophages polarization during the course of obesity, thereby accelerating adipose tissue inflammation (9). M1 macrophages secrete pro-inflammatory cytokines including TNF-α, IL-6, and IL-1β, that act as main effectors of impaired adipocyte function and inflammatory signals. On the one hand, the pro-inflammatory factors derived from M1 macrophages like TNF-α act on the receptors in hypertrophied adipocytes, thereby inducing pro-inflammatory cytokine production such as monocyte chemotactic protein-1 (MCP-1) and adipocyte lipolysis through nuclear factor-κB (NF-κB)-dependent and -independent [possibly members of mitogen-activated protein kinases (MAPK)-dependent] mechanisms, respectively (10). A series of processes described above promote the decomposition of adipocytes and the continuous production of free fatty acids (FFA). Dysregulation of adipocytokines and overproduction of FFA will result in ectopic lipid accumulation and lipotoxicity such as endothelial dysfunction (atherosclerosis), insulin resistance (diabetes), and non-alcoholic steatohepatitis (11). On the other hand, saturated fatty acids emerged from adipocytes activate Toll-like receptor 4 (TLR4) signaling in macrophages and promote the release of inflammatory factors (12). A deficiency of TLR4 could protect against obesity-induced M1 polarization and adipose tissue inflammation (12). This finding indicates that the vicious cycle established among adipocytes and ATMs augments chronic inflammatory changes (13) and the inhibition of chronic inflammation is a significant target in the treatment of insulin resistance and metabolic syndrome.

Mushrooms are widely grown in nature, and many of them have been traditionally used as medicinal foods in Asian countries (14, 15). Mushroom *Phellinus linteus* (“*Sanghuang*” in Chinese) is a popular medicinal polypore used throughout China, Japan, and Korea (16) and plays a physiological function in resistive effects of oxidize, germ, and tumor, as well as reducing the blood sugar and lipemia and improving immunity (17–19). More importantly, no apparent adversities after its consumption have been reported. While 15 *sanghuang* mushroom species have been found in the world, only some of them were found to display anti-inflammatory, antioxidant and anti-carcinogenic activities (16, 19, 20). *Inonotus sanghuang* (*I. sanghuang*) is one species of *sanghuang* mushroom and a white-rot fungus in the family of *hymenochaetaceae*. Ethanol extract of *I. sanghuang* mycelia produced from liquid fermentation scavenged DPPH and hydroxyl radicals has been shown to have anti-oxidant activity due to the existing phytochemicals (polyphenolics), such as rutin, eriodictyol, naringenin, and sakuranetin (21). Our recent *in vitro* study has shown that the anti-oxidant, anti-proliferative, and anti-microbial activities have been found in *I. Sanghuang* extract (polyphenols) from another *Sanghuang* species, wild *I. Sanghuang* from the Aershan Region of Inner Mongolia (Inner Mongolia, China) (22). However, there are no studies exploring the anti-inflammatory and immunomodulating properties of wild *I. Sanghuang* from the Aershan Region of Inner Mongolia and its use for the prevention/treatment of inflammation-related diseases.

In the present study, we investigated the anti-inflammatory property of *I. sanghuang* extract (ISE) on RAW264.7 macrophages and then explored the effect of ISE on the crosstalk between RAW264.7 macrophages and 3T3-L1 adipocytes using *in vitro* cell coculture models.

## Materials and Methods

### Chemicals

*I. sanghuang* was collected from the Aershan Region of Inner Mongolia (Inner Mongolia, China) and *I. sanghuang* extract (ISE) was prepared as described previously (22). ISE polyphenols from ethyl acetate fraction of *I. sanghuang* powder was prepared and have been characterized as described previously to identify 6 compounds, namely, rutin, quercetin, quercitrin, icarisid II, isorhamnetin and chlorogenic acid, which have been suggested to have potent anti-oxidant, anti-proliferative and anti-microbial activities (22). Thus, we use ethyl acetate fraction (EAF) as *I. sanghuang* extract (ISE) for this study. Concentrated ISE was stored at −20°C until further use. A stock solution of ISE dissolved in DMSO at 10 mg/mL was stored at −80°C and diluted with culture medium to the appropriate working concentrations immediately prior to use.

### Cell Cultures

The RAW264.7 macrophage cell line was provided by J. Jin (Zhejiang University, Hangzhou, China) and maintained in DMEM supplemented with penicillin (100 U/mL)-streptomycin (100 μg/mL) and 10% heat-inactivated fetal bovine serum in a humidified 5% CO_2_ atmosphere at 37°C. The method of 3T3-L1 adipocyte differentiation was performed as described previously (23). Briefly, 3T3-L1 preadipocytes (Saierbio Inc., Tianjin, China) were cultured in 24-well plates (2.5 × 10^5^ cells/well) in DMEM with 10% calf serum, 100 U/mL penicillin, and 100 μg/mL streptomycin at 37°C under a humidified 5% CO_2_ atmosphere. Two days after the preadipocytes post confluency, use 3-Isobutyl-1-Methylxanthine (IBMX, 0.5 mM), dexamethasone (1.0 μM) and insulin (10 μg/mL) in 10% FBS/DMEM to stimulate the cells. Twelve to twenty days later, a large amount of red lipid droplets were observed by oil red O staining and the cells were used as hypertrophied 3T3-L1 adipocytes (Supplemental Figure 1). Adipocytes and macrophages were cocultured in a contact system as previously described (23). Briefly, RAW264.7 macrophages (2.5 × 10^5^ cells/well) were plated into dishes with serum-starved and hypertrophied 3T3-L1 adipocytes. The coculture was incubated in serum-free DMEM for 24 h. RAW264.7 macrophages and 3T3-L1 adipocytes were cultured separately under the same conditions for contrast. Different concentrations of ISE were administrated meanwhile as that of coculture. The supernatants were collected and stored at −20°C until further measurements. At the same time, RAW264.7 macrophages were seeded in 24-well plates (2.5 × 10^5^ cells/well) and treated with either 1 μg/mL LPS or 3T3-L1 adipocyte conditioned medium (L1CM) for 30 min or 24 h with different concentrations of ISE. The hypertrophied 3T3-L1 adipocytes were cultured in serum-free medium for 12 h, and then collected the supernatants and designated as the L1CM and stored at −20°C until use. As for the effect of ISE on the viability of RAW264.7 macrophages, RAW264.7 were transferred into 96-well plates at 1 × 10^4^ cells/well and incubated with ISE (final concentrations: 0.5, 1.0, 2.0, and 4.0 μg/mL) for 24 h. The wells were incubated in the absence of ISE as the control group and CCK-8 (Dojindo, Kumamoto, Japan) was added to each well for cell staining for another 4 h according to the manufacturer's instruction. The cell viability was calculated as a percentage of control, which was considered as 100%, according to the formula: (A _sample_-A _blank_)/(A _control_-A _blank_) × 100. Plates were read at OD_450_ in a microplate reader (BIO-TEK Instruments, Winooski, VT, USA).

### Measurement of MCP-1, TNF-α, IL-6, and NO

Cell-free supernatants were collected to determine the MCP-1 (Biolegend, San Jose, CA), TNF-α, and IL-6 (both from eBioscience, San Diego, CA) using ELISA kit assay. In addition, nitrite as the end-point of NO generation from activated macrophages was measured by determining NO_2_-concentration in the culture supernatant. Briefly, add 100 μL of Griess reagent (1% sulfanilamide and 0.1% N-[1-naphthyl]-ethylenediamine dihydrochloride in 5% phosphoric acid) into 100 μL samples of medium. The concentration of NO_2_ was calculated by extrapolating a NaNO_2_ standard curve.

### Immunoblotting

After being pretreated with ISE, RAW264.7 macrophages were stimulated with LPS or L1CM for 30 min, and then harvested in RIPA lysis buffer containing 50 mM Tris-HCl (pH 7.4), 150 mM NaCl, 1% NP40, 1 × protease inhibitor cocktail (Roche Applied Science, Indianapolis, IN), and 1 × phosphatase inhibitor cocktail (Sigma-Aldrich). Proteins were resolved in 7.5% acrylamide gels and then transferred to nitrocellulose membranes. The membrane was blocked with 5% non-fat milk in Tris-buffered saline before being incubated, respectively with specific primary antibodies for the following proteins: IκB-α (1:1000), phosphor-p44/p42 (Thr202/Tyr204) (p-ERK) (1:1000), phosphor-p38 (Thr202/Tyr204) (p-p38) (1:1000), and Phospho-SAPK/JNK (Thr183/Tyr185) (p-JNK) (1:1000), phosphor-STAT3 (p-STAT3) (1:1000), ERK (1:1000), JNK (1:1000), p38 (1:1000), STAT3 (1:1000) (all from Cell Signaling Technologies, Danvers, MA), and β-actin (1:5000, Sigma-Aldrich). The membranes were next incubated with horseradish peroxides (HRP)-conjugated secondary antibodies followed by exposure to enhanced chemiluminescent reagents (Millipore, Burlington, MA).

### Macrophage Migration Assay

Migration assays were performed using Transwell inserts with a 5 μm membrane pore size (Corning, NY). The L1CM from fully differentiated 3T3-L1 adipocytes was transferred to 24 well plates containing inserts. The RAW264.7 macrophages were pre-incubated with or without ISE for 1 h. Then, the RAW264.7 macrophages were seeded onto the inserts at 5 × 10^4^ cells/well. After migrating for 4 h at 37°C, any non-migrated cells were wiped off with a cotton swab, and the cells adhering to the underside of the membrane were fixed with 4% paraformaldehyde for 20 min. The membranes were then washed with PBS. The number of migrated cells in twelve random microscopy fields per membrane was counted at 400 × magnification.

### Statistical Analysis

All data were presented as the means ± SD. The statistical significance of any difference in each parameter among treatment was evaluated by one-way analysis of variance (ANOVA) followed by Tukey's test using Prism 6.0 software. Significance level was set at *P* < 0.05.

## Results

### ISE Inhibited the Production of Inflammation Mediators Released by LPS-Activated RAW264.7 Macrophages

**Figure 1:**
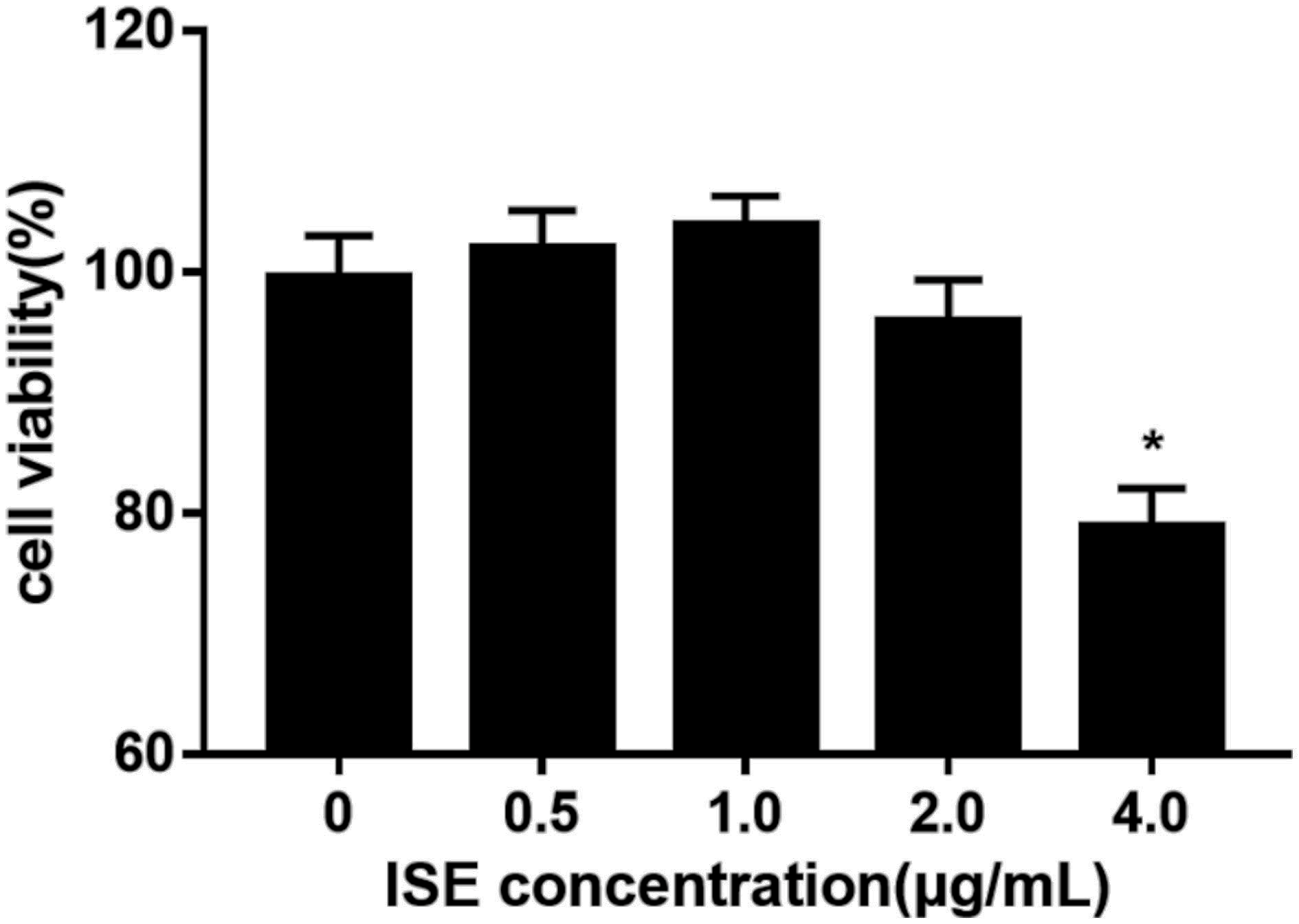
Cell viability of RAW264.7 macrophages pretreated with different concentrations of ISE. RAW264.7 macrophages were incubated with ISE in the indicated concentrations for 24 h and cell viability was determined using CCK-8 kit as described in “*Materials and Methods*” section. The values are means ± SD, *n* = 6, ^*^P < 0.01.

To investigate whether ISE affects the production of pro-inflammatory mediators from macrophages in obesity-related environments, RAW264.7 cells were treated with different doses of ISE in the presence or absence of LPS. We first determined the effect of ISE at different doses on cell viability. ISE at 4.0 μg/mL, not 0.5, 1.0 and 2.0 μg/mL, used according to the anti-oxidant activity of ISE as previously described (22), had the toxicity on cell viability (Figure 1). Thus, we used ISE concentrations at 0.5, 1.0, and 2.0 μg/mL in the subsequent experiments to determine the working mechanisms of ISE.

**Figure 2:**
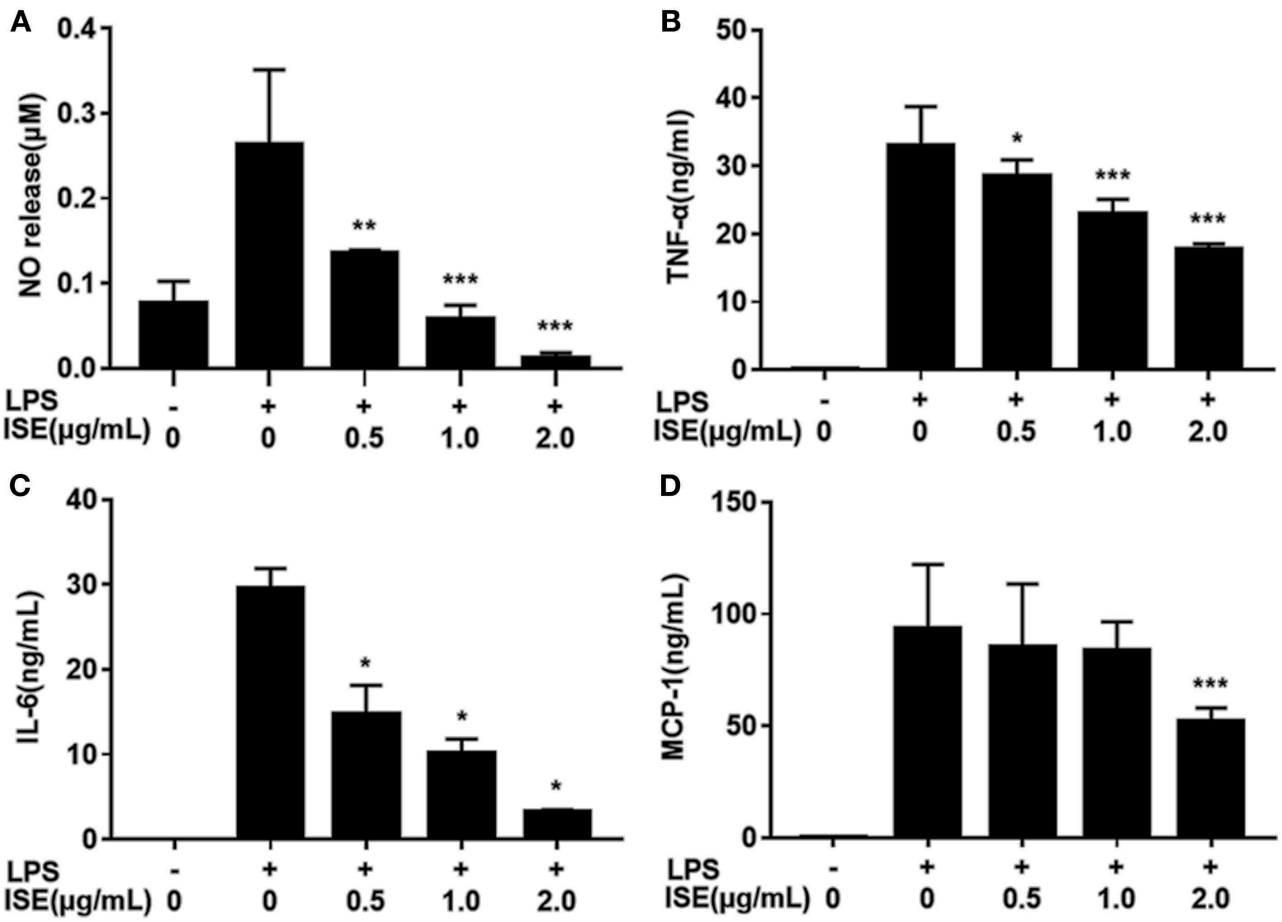
Effects of ISE on RAW264.7 macrophage activation by LPS. RAW264.7 macrophages were pretreated with different concentrations of ISE for 1 h and then were incubated with LPS at 1.0 μg/mL for 24 h. Cell-free supernatants were collected to determine the concentrations of NO **(A)**, TNF-α **(B)**, IL-6 **(C)**, and MCP-1 **(D)** as described in “*Materials and Methods*” section. The values are means ± SD of 6 samples. Statistical comparisons were made with each vehicle control. ^*^P < 0.05, ^**^P < 0.01, ^***^P < 0.001.

Next we determined the effect of ISE on inflammation mediators produced by LPS-activated RAW264.7 macrophages, which are M1 macrophages induced by LPS or pro-inflammatory mediators and produce significant amounts of pro-inflammatory cytokines, such as TNF-α, IL-6, and NO (24, 25). ISE administration decreased NO production in a dose-dependent manner (IC_50_ = 0.52 μg/mL) (Figure 2A). In addition, the secretion of TNF-α (IC_50_ = 2.22 μg/mL) (Figure 2B), and IL-6 (IC_50_ = 0.43 μg/mL) (Figure 2C) were inhibited dose-dependently by ISE administration in LPS-activated macrophages. However, ISE only reduced the MCP-1 secretion (IC_50_ = 2.24 μg/mL) at 2.0 μg/mL (Figure 2D).

### ISE Reduced the Level of Inflammation Mediators Released by RAW264.7 Macrophage Stimulated by 3T3-L1 Adipocyte Conditioned Medium

**Figure 3:**
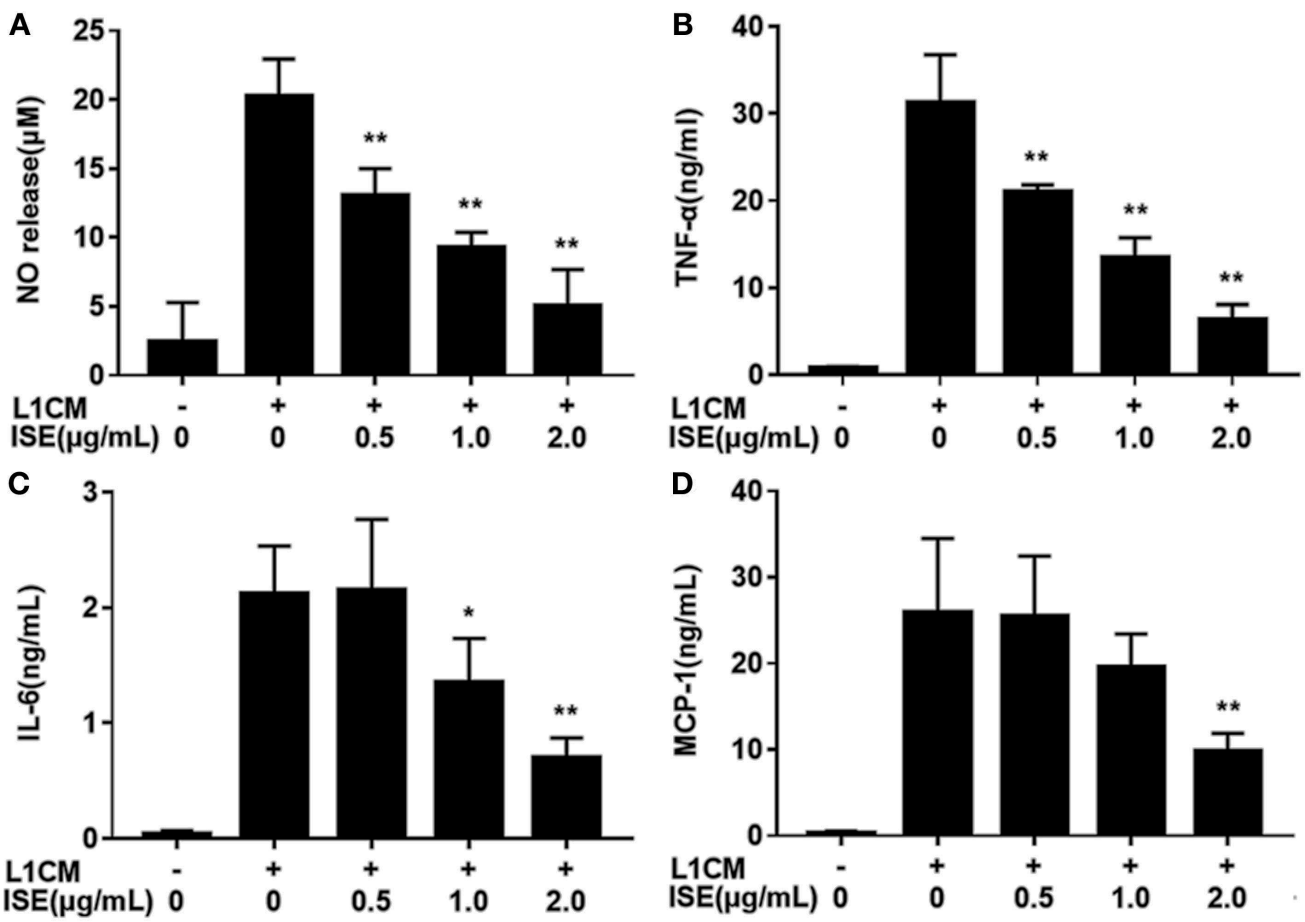
Effects of ISE on inflammation in RAW264.7 macrophages activated by conditioned medium of hypertrophied 3T3-L1 adipocytes. RAW264.7 macrophages were pretreated with different concentrations of ISE for 1 h and then were incubated with conditioned medium of hypertrophied 3T3-L1 adipocytes (L1CM) for 24 h. Cell-free supernatants were collected to determine the concentrations of NO **(A)**, TNF-α **(B)**, IL-6 **(C)**, and MCP-1 **(D)** as described in “*Materials and Methods*” section. The values are means ± SD of 6 samples. Statistical comparisons were made with each vehicle control. ^*^P < 0.05, ^**^P < 0.01.

To investigate whether ISE affected the secretion of pro-inflammatory cytokines from macrophages in obesity-related environments, RAW264.7 macrophages were co-treated with ISE and L1CM to induce an obesity-related inflammatory reaction. Pro-inflammatory mediators NO (Figure 3A), TNF-α (Figure 3B), IL-6 (Figure 3C), and MCP-1 (Figure 3D) production apparently increased in L1CM-activated models (IC_50_ = 0.84, 0.82, and 1.40 μg/mL, respectively). In the model with L1CM, the suppressive property of ISE was observed as NO, TNF-α, and IL-6 in a dose-dependent manner. In accordance with MCP-1 released by LPS-activated macrophages, only ISE at 2.0 μg/mL decreased MCP-1 production (IC_50_ = 1.62 μg/mL).

### ISE Administration Decreased the Pro-Inflammatory Cytokine Level in Cocultures of 3T3-L1 Adipocytes and RAW264.7 Macrophages

**Figure 4:**
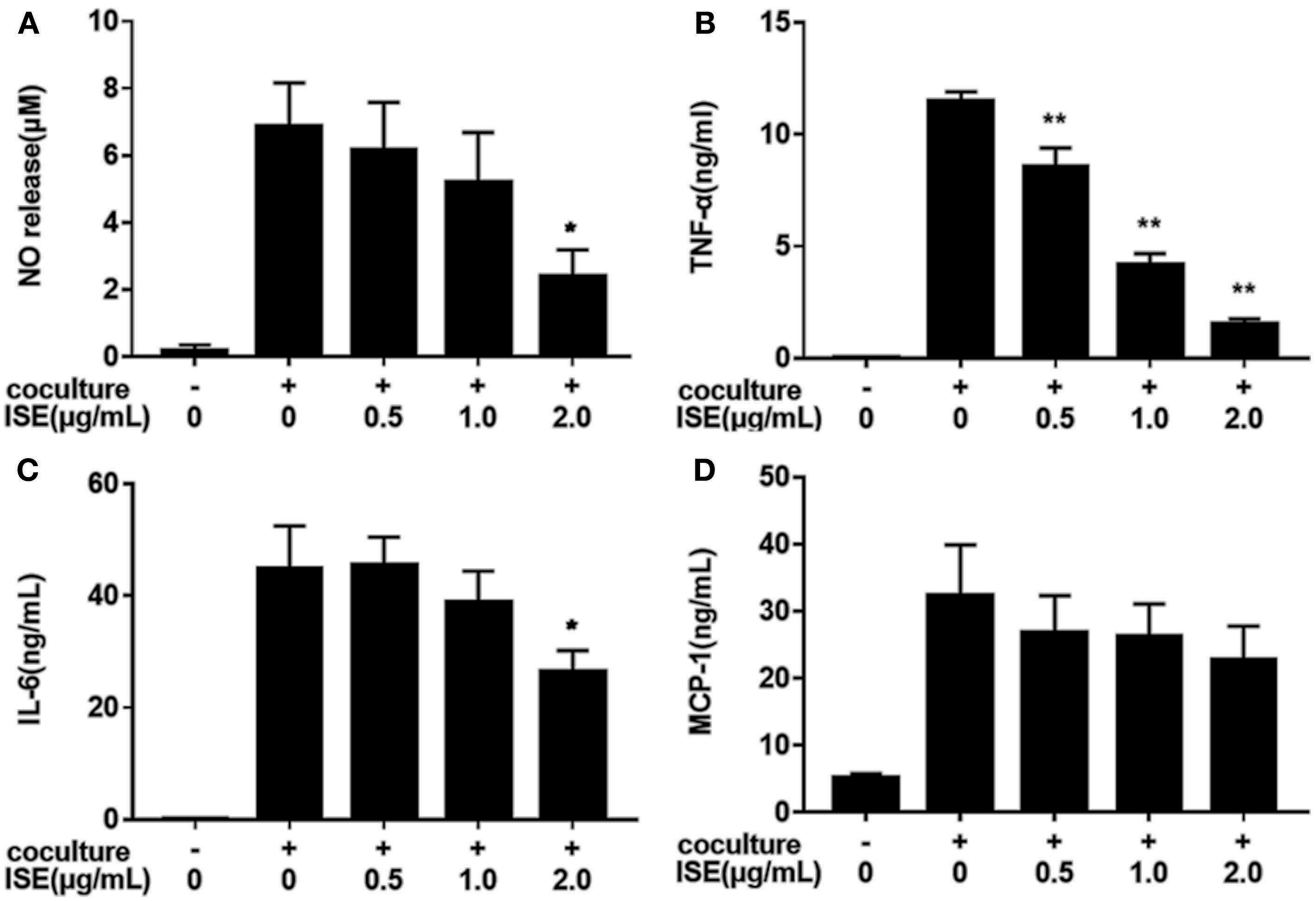
Effects of ISE on inflammation in RAW264.7 macrophages cocultured with differentiated 3T3-L1 adipocytes. RAW264.7 macrophages were pretreated with different concentrations of ISE for 1 h and then were incubated with differentiated 3T3-L1 adipocytes for 24 h. Cell-free supernatants were collected to determine the concentrations of NO **(A)**, TNF-α **(B)**, IL-6 **(C)**, and MCP-1 **(D)** as described in “*Materials and Methods*” section. The values are means ± SD of 6 samples. Statistical comparisons were made with each vehicle control. ^*^P < 0.05, ^**^P < 0.01.

We next examined whether ISE treatment could suppress inflammatory changes in a coculture system composed of 3T3-L1 adipocytes and RAW264.7 macrophages. In this model, 3T3-L1 adipocytes or RAW264.7 macrophages were cultured separately and treated with ISE by the same method that we used in the coculture system. As shown in Figure 4, ISE treatment dose-dependently suppressed the production of NO (IC_50_ = 1.55 μg/mL) (Figure 4A), TNF-α (IC_50_ = 0.80 μg/mL) (Figure 4B) and IL-6 (Figure 4C) (IC_50_ = 2.30 μg/mL), but did not affect MCP-1 (Figure 4D) in the coculture system. These data indicate that ISE treatment has the ability to block the release of inflammatory mediators in the coculture system.

### The Anti-inflammatory Effects of ISE Were Caused by Inhibition of MAPK and STAT Activity

TLR4 is the main pathway for stimulating inflammatory responses. To investigate the molecular mechanism of the anti-inflammatory effects of ISE, we examined TLR4 signaling pathway in RAW264.7 macrophages.

**Figure 5:**
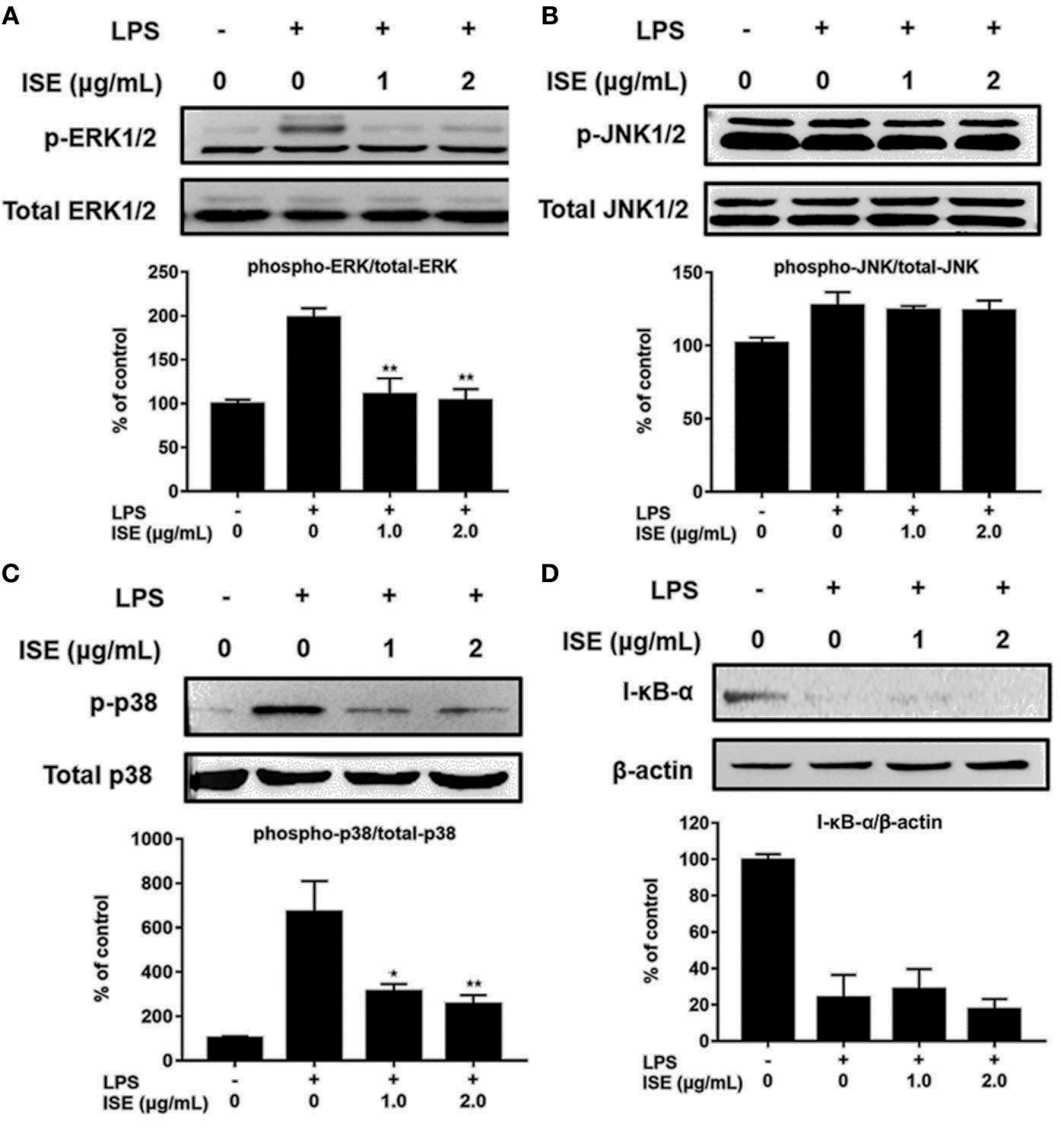
Effects of ISE on LPS-induced MAPK and NF-κB signals. RAW264.7 macrophages were pretreated with different concentrations of ISE for 1 h and then stimulated by 1.0 μg/mL LPS for 30 min. Total cell lysates were extracted, and then western blotting using specific antibodies was used for the detection of phosphorylated and total forms of three MAPK molecules, ERK **(A)**, JNK **(B)**, p38 **(C)**, and I-κB-α **(D)**. The value of a control was set at 100%, and the relative value was presented as fold induction to that of the control, which was normalized to total MAPK or β-actin. The values are means ± SD, *n* = 3. Statistical comparisons were made with each vehicle. ^*^P < 0.01, ^**^P < 0.001.

**Figure 6:**
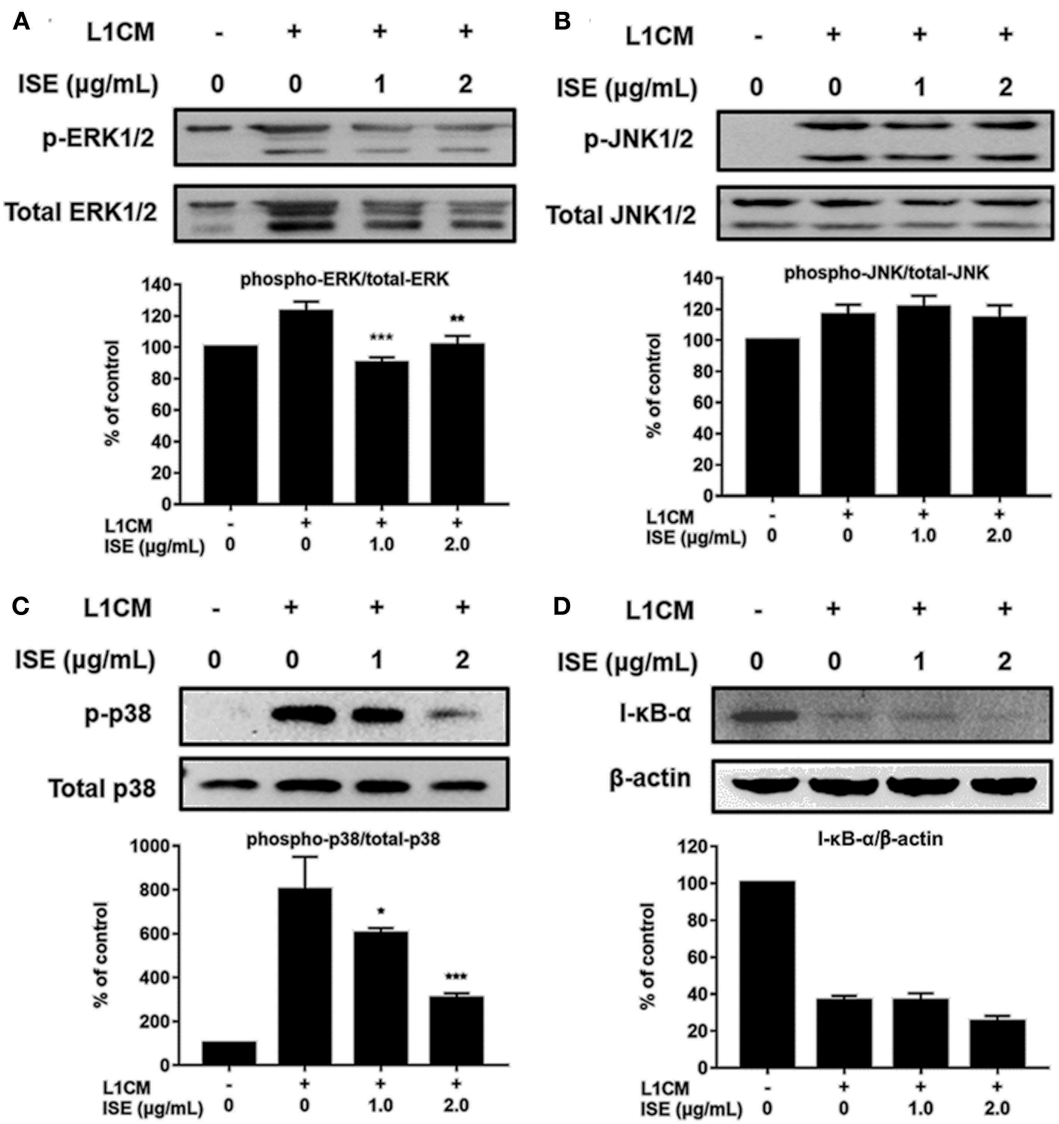
Effects of ISE on 3T3L1 CM-induced MAP kinase and NF-κB signals. RAW264.7 macrophages were pretreated with different concentrations of ISE for 1 h and then stimulated by 3T3-L1 CM (L1CM) for 30 min. Total cell lysates were extracted to detect the levels of phosphorylated ERK **(A)**, JNK **(B)**, p38 **(C)**, and I-κB-α **(D)**. The value of a control was set at 100%, and the relative value was presented as fold induction to that of the control, which was normalized to total ERK, JNK, p38 or β-actin. The values are means ± SD, *n* = 3. Statistical comparisons were made with each vehicle controls. ^*^P < 0.05, ^**^P < 0.01, ^***^P < 0.001.

TLR4 signaling stimulated by LPS or L1CM results in the activation of NF-κB and MAPK family including ERK, JNK, and p38 MAPK, which drive the upregulation of pro-inflammatory genes (26, 27). To characterize the mechanism underlying the suppression of pro-inflammatory mediators by ISE, we used Western blotting to measure the phosphorylation of ERK, JNK, p38, and the degradation of I-κB-α. Stimulation with LPS or L1CM increased the phosphorylation of ERK (Figures 5A, 6A), JNK (Figures 5B, 6B), p38 (Figures 5C, 6C), and enhanced the degradation of I-κB-α (Figures 5D, 6D). While ERK and p38 activation were significantly suppressed by ISE, the phosphorylation of JNK was not impacted by ISE in LPS- or L1CM- activated macrophages. Furthermore, ISE treatment could not prevent the degradation of I-κB-α, which suggests that NF-κB signals may not be involved in the inhibitory effects of ISE on pro-inflammatory mediator release.

**Figure 7:**
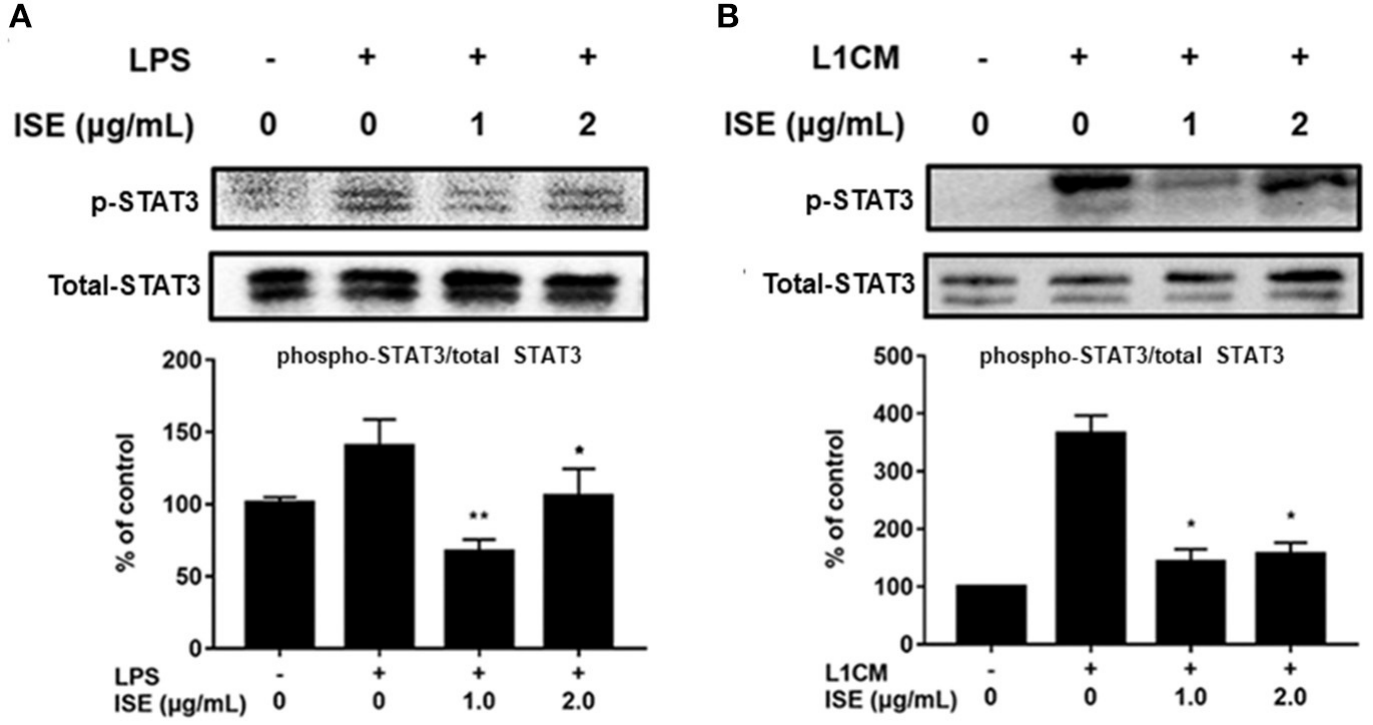
Effects of ISE on STAT3 signal. RAW264.7 macrophages were pretreated with different concentrations of ISE for 1 h and then stimulated by 1.0 μg/mL LPS or 3T3-L1 CM for 30 min. Total cell lysates were extracted, and then western blotting using specific antibodies was used to determine the expression of p-STAT3 and STAT3 in LPS **(A)** and 3T3-L1 CM (L1CM) stimulation **(B)**, respectively. The value of a control was set at 100%, and the relative value was presented as fold induction to that of the control, which was normalized to total STAT3. Statistical comparisons were made with each vehicle controls. The values are means ± SD, *n* = 3. ^*^P < 0.05, ^**^P < 0.01.

STAT signals are also important transcriptional activators that regulate cytokine transcription (28). Inflammatory cytokine IL-6 is one of the active mediators of STAT3, which transmits signals through its receptor IL-6 and activates Janus kinase, which in turn activates the STAT family, including the phosphorylation and activation of STAT3 (29). To further explore the effect of ISE on the decrease of pro-inflammatory mediators through JAK/STAT3 signaling pathway in the obesity-related environment, we measured the protein expression level of p-STAT3 and STAT3 in LPS (Figure 7A) and L1CM (Figure 7B), respectively. Data showed that the phosphorylation of STAT3 was markedly suppressed by ISE in LPS- and L1CM- activated models.

### ISE Blocked the LICM-Stimulated Migration of RAW264.7 Macrophages

**Figure 8:**
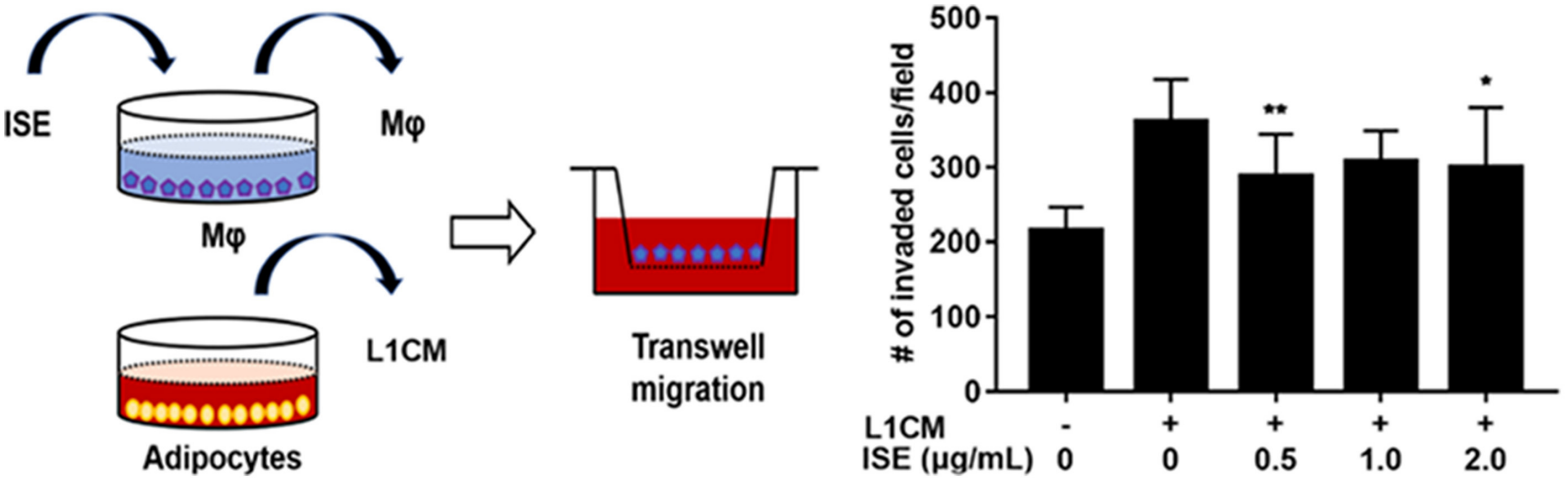
Effects of ISE on macrophage motility. (**Left**) Procedures of the macrophage migration experiments. RAW 264.7 macrophages were treated with vehicle or ISE (0.5, 1.0, 2.0 μg/mL) for 1 h and the detached cells were used for migration assay in the presence of DMEM or L1CM. (**Right**) Migrated RAW264.7 macrophages were quantified as described in “*Materials and Methods*” section. The values are means ± SD, *n* = 12. ^*^P < 0.05, ^**^P < 0.01. Mφ, RAW264.7 macrophages.

To examine the potential of ISE to limit the motility of macrophages, RAW264.7 macrophages were preincubated with different concentrations of ISE ahead of L1CM incubation and compared with cells incubated with L1CM alone. As shown in Figure 8, L1CM induced an obvious migration of macrophages. While this process was suppressed by ISE.

## Discussion

**Figure 9:**
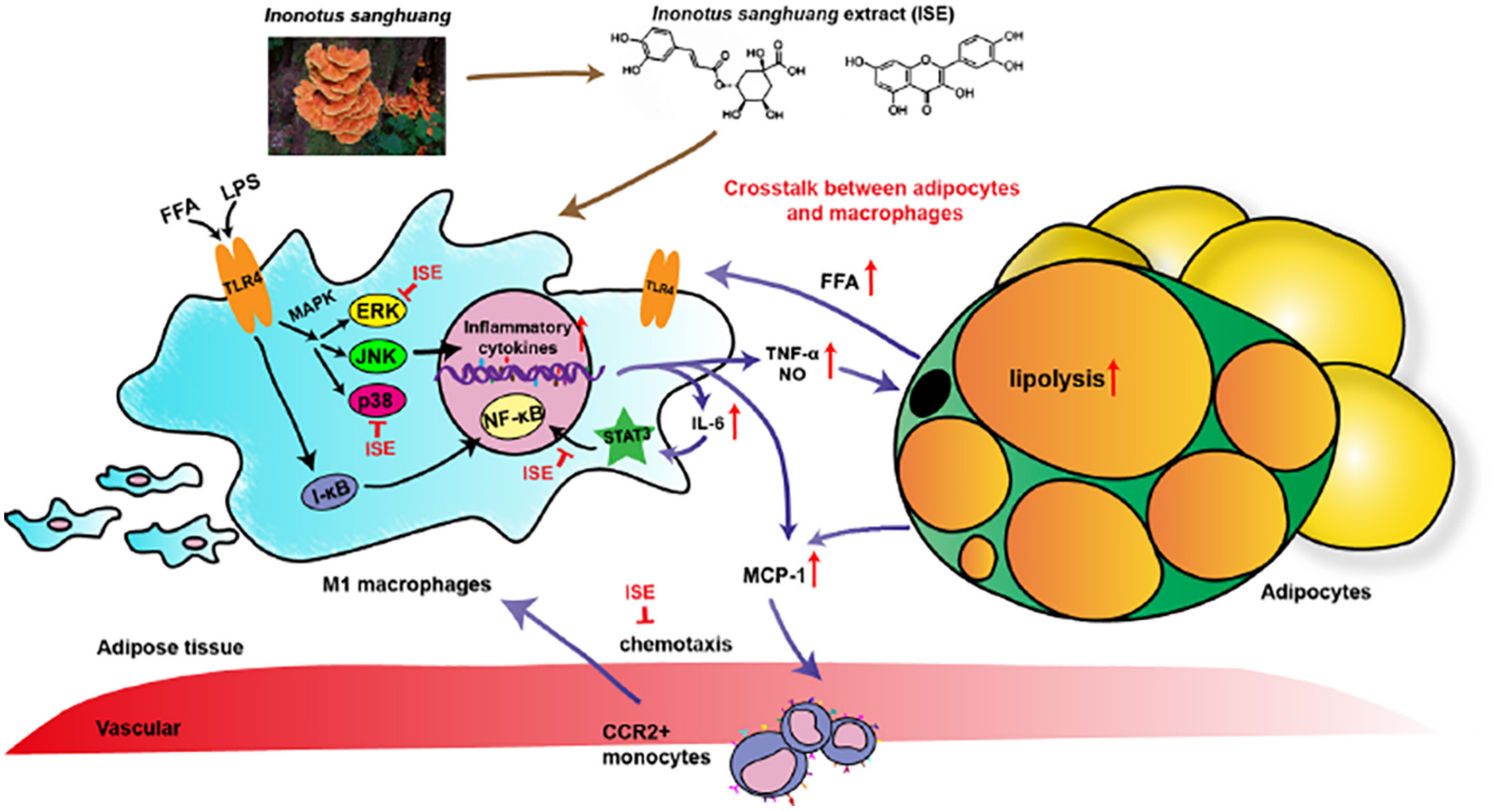
Schematic models of molecular targets affected by ISE to attenuate inflammatory signaling pathways. On the one hand, LPS- and free fatty acid (FFA)-induced inflammatory responses are regulated by both NF-κB and MAPK signaling pathways. In activated macrophages, ISE decreases pro-inflammatory cytokine production via inhibiting the ERK and p38 MAPK pathways, not NF-κB signals. On the other hand, ISE markedly suppresses the phosphorylation of STAT3 to reduce cytokines transcription. Subsequently, the lipolysis of adipocytes is suppressed and proinflammatory M1 macrophages are less recruited via the MCP-1-CCR2 pathway. And the decrease of inflammatory cytokine IL-6 further restrains the IL-6/STAT3 activation and the inflammatory mediator production. In a word, treatment with ISE reduces the levels of these pro-inflammatory mediators, thereby disrupting the crosstalk between macrophages and adipocytes in a coculture. ISE apparently exerted anti-inflammatory ability, possibly diminishing the obesity-induced inflammatory diseases.

*I. sanghuang*, as a popular medicinal polypore used throughout Southeast Asia, was reported diffusely for its effects of anti-oxidant, anti-tumor, anti-microbial, lipid-lowering effect (30). Evidence has shown that various ingredients of *I. sanghuang*, such as polysaccharides (18, 31), had anti-inflammatory activity. However, the anti-inflammatory activity of polyphenols extracted from *I. sanghuang* has not been reported. The present study showed that ISE polyphenols directly inhibits pro-inflammatory cytokines NO, TNF-α, and IL-6 released by RAW264.7 macrophages activated by LPS, L1CM, and cocultured with differentiated 3T3-L1 adipocytes. These effects might be mediated via suppressing p-ERK- and p-p38- MAPK and STAT3 signaling pathways (Figure 9). These data indicate that ISE polyphenols have the ability to interfere the crosstalk between macrophages and adipocytes and inhibit obesity-induced adipose inflammation.

It's beyond dispute that obesity-induced adipose inflammation by paracrine interactions between adipocytes and adipose-infiltrating macrophages plays a causative role in insulin resistance and metabolic dysfunction in obesity and is characterized by abnormal secretion of pro-inflammatory cytokines in white adipose tissue (32). On the one hand, adipose tissue promotes macrophages to secrete inflammatory mediators by releasing FFA and to accumulate with chemotaxis in adipose tissue by releasing MCP-1. On the other hand, inflammatory cytokines from ATMs could accelerate the secretion of FFA from adipose tissue. In other words, macrophages markedly infiltrate into obese adipose tissue and establish an inflammatory paracrine loop with adipocytes. From what has been discussed above, it's expected to reduce the chronic low-grade inflammatory response in obesity by cutting off the interaction between macrophages and adipocytes mediated by reducing the secretion of pro-inflammatory mediators.

*In vitro*, coculture of highly differentiated 3T3-L1 adipocytes and RAW264.7 macrophages has been used as the model of adipose inflammation in which pro-inflammatory cytokine genes and proteins such as MCP-1, IL-6, and TNF-α are significantly upregulated (33). Several polyphenols have been reported to be able to reduce secretion of pro-inflammatory indicators in the coculture model (34, 35). However, ISE polyphenols have not been tested. Additionally, TLR4, the receptor of LPS, which could activate the NF-κB and MAPK signaling pathways, is expressed in various cells, including adipocytes and macrophages (36, 37). It can be activated by LPS or endogenous saturated FFA and initiate potent downstream inflammatory responses in obese adipose tissue (12). Furthermore, this is supported by a study showing that TLR4 deficiency can prevent insulin resistance in lipid-infused male mice (37). Thus, inhibition of TLR4 signaling has been shown as an attractive therapeutic strategy for treatment of obesity-induced insulin resistance and adipocytes mediated chronic inflammation.

In this study, ISE suppressed production of pro-inflammatory mediators in LPS- or L1CM-activated RAW264.7 macrophages and a coculture system with 3T3-L1 adipocytes and RAW264.7 macrophages. We found that ISE dose-dependently may directly suppress production of NO, TNF-α, IL-6, and MCP-1 (IC_50_ = 0.52, 2.22, 0.43, and 2.24 μg/mL, respectively) in the single culture of RAW264.7 macrophages activated by LPS. LPS from gram-negative intestinal microbiota is an early factor recruiting a signals through TLR4 to induce secretion of pro-inflammatory cytokines and then triggering metabolic diseases induced by a high-fat diet (38, 39). Our and these data suggest that ISE may have beneficial effects on obesity-related diseases. Current data supported that the production of pro-inflammatory mediators NO, TNF-α, IL-6, and MCP-1 (IC_50_ = 0.84, 0.82, 1.40, and 1.62 μg/mL, respectively) could be significantly suppressed by ISE in L1CM-activated RAW264.7 macrophages in a dose-dependent manner. It has been reported that FFA secreted from the L1CM which is derived from hypertrophied adipocytes is the key factor to enhance the expression of TNF-α in ATMs (40). Thus, these data indicate that 3T3-L1 CM could activate RAW264.7 macrophages to induce inflammatory response possibly via adipocytes secreting FFA and other cytokines. Moreover, our data suggest that ISE might be able to reduce pro-inflammatory mediators NO, TNF-α, and IL-6 (IC_50_ = 1.55, 0.80, and 2.30 μg/mL, respectively) and then promote inflammatory changes in obese adipose tissue. However, whether it is true needs to be investigated. In addition, ISE treatment failed to promote changes in MCP-1 in the coculture system. It's well known that MCP-1 plays an important role in both potently recruiting macrophages into adipose tissue and determining a more inflammatory M1 macrophage phenotype (41), suggesting that MCP-1 is secreted by 3T3-L1 adipocytes from the most part rather than by RAW264.7 macrophages. Thus, we speculate that anti-inflammatory effects of ISE might have more impact on the activated macrophages in adipose tissue. Finally, we noted that among different activated macrophages, the mean IC_50_ values of ISE toward inflammatory mediators NO, TNF-α and IL-6 was at least 2.0 μg/mL less than the other mediator MCP-1, suggesting that the inhibitory effects of ISE on the interaction between adipocytes and macrophages might mainly be caused by suppressing pro-inflammatory mediators.

Previous studies have shown that some species of *sanghuang* exert anti-inflammatory effects through inhibiting the signals of NF-κB and MAPKs (e.g., ERK and p38) (42–44). In addition, polyphenols have been demonstrated to suppress inflammatory responses by blocking the ERK and NF-κB pathways in microglia and inhibit production of pro-inflammatory cytokines TNF-α, IL-1β, and IL-6 (45). Current study found that ISE could down-regulate MAPKs, not NF-κB signaling triggered by L1CM-activated macrophages. Notably, activation of ERK and p38, not JNK, was inhibited by ISE, and these two are considered to be the major mechanism that accounts for the suppression of pro-inflammatory mediator secretion by ISE.

Polyphenols are the secondary metabolites of mushrooms in the genus *Inonotus*. In the current study, 6 polyphenols such as quercetin, rutin, quercitrin, icarisid II, isorhamnetin and chlorogenic acid are contained in ISE which could exert anti-oxidant, anti-proliferative, and anti-microbial activities *in vitro* (22). Quercetin has been shown to be able to inhibit the MAPK signals in adipocytes, macrophages, lipid accumulation and obesity-induced inflammation in the animal models (46). Although rutin has been reported to have no effect on lipid accumulation in 3T3-L1 adipocytes, it can attenuate obesity through activating brown fat and then increasing energy expenditure (47). Furthermore, quercitrin (48), Icaritin II (49), isorhamnetin (50), and chlorogenic acid (51) have also been reported to have anti-inflammatory activities in macrophages or animal models. These reports and current data indicate that the attenuation of ISE on inflammatory response in macrophages or crosstalk between macrophages and adipocytes be mediated, at least in part by *I. sanghuang* polyphenols. However, which compound is involved in this effect needs to be investigated.

In this study, we also demonstrated that ISE inhibited STAT3 phosphorylation, as one of the pathways to suppress production of pro-inflammatory mediators in activated macrophages. Polyphenols could reduce production of inflammatory factors via suppressing IL-6/STAT3 signaling pathway (52, 53). In addition, polyphenols treatment could prevent the pro-inflammatory effect on macrophages by potentially inhibiting STAT3 activation (54). Since ISE treatment could inhibit IL-6 released by macrophages activated by LPS or L1CM, we speculate that ISE might have the ability to affect IL-6/STAT3 signals. As expected, the activation of STAT3 was markedly inhibited by ISE. Therefore, our data suggest that the anti-inflammatory effect of ISE may be mediated, not only by inhibiting Erk and p38 MAPK signaling, but also by affecting the reduction of IL-6 and inhibiting IL-6/STAT3 signaling pathway. In addition, whether ISE administration has benefits in obesity-related diseases and the underlying mechanism need to be investigated.

As described above, activated macrophages infiltrating into adipose tissue are critical source of pro-inflammatory mediators, which subsequently cause systemic insulin resistance and obesity-related complications. Therefore, preventing the infiltration of macrophages into adipose tissue could ameliorate obesity-related inflammation and provide a therapeutic target for obesity-related complications. However, the mechanisms that initiate macrophages recruitment to adipose tissue remain not fully elucidated, but it presumably involves increased secretion of chemotactic factors, in particular chemokine MCP-1 secreted from adipocytes (55). This study gives rise to the question of how ISE prevents macrophages migration, while we couldn't supply an immediate answer to this important question at present. Our data showed that ISE didn't inhibit secretion of MCP-1 from cocultures; in other words, ISE presumably inhibits macrophage migration by acting on macrophages rather than adipocytes. Previous studies have demonstrated that MCP-1 induces macrophage migration largely via MAPKs signaling (56). In addition, LPS increases macrophage motility (57), suggesting the possibility that ISE could impair the ability of macrophages to migrate in response to MCP-1 by inhibiting TLR4 activity. Researchers have also found that polyphenols from tea could significantly suppress proliferation, migration, and invasion in melanoma by TLR4 inhibition (58). Moreover, macrophages treated with butein (a polyphenol of vegetal origin) exhibited a reduced ability to migrate toward 3T3-L1 CM (59). According to the above, this study implies that ISE at higher concentrations has the potential to block ATMs recruitment in the adipose tissue by TLR4 or any other signaling pathways inhibition, which needs to be clarified further.

In conclusion, we demonstrated that ISE has the ability to inhibit the crosstalk between adipocytes and macrophages, mediated by attenuating inflammatory responses produced by macrophages (Figure 9). Our results suggest that ISE polyphenols may be valuable as a functional medicinal food in terms of ameliorating chronic inflammation in obese adipose tissue and improving obesity-related insulin resistance and complications.

## Author Contributions

JW, MZ, WP, and YZ contributed to the design of the study. MZ, YX, XS, and KL, performed the experiments. MZ analyzed the data. MZ and JW wrote or critically revised the manuscript. All authors read and approved the final manuscript.

### Conflict of Interest Statement

The authors declare that the research was conducted in the absence of any commercial or financial relationships that could be construed as a potential conflict of interest.
