# Corrigendum: *Inonotus sanghuang* Polyphenols Attenuate Inflammatory Response *Via* Modulating the Crosstalk Between Macrophages and Adipocytes

**DOI:** 10.3389/fimmu.2021.633354

**Published:** 2021-01-28

**Authors:** Mengdi Zhang, Yu Xie, Xing Su, Kun Liu, Yijie Zhang, Wuyan Pang, Junpeng Wang

**Affiliations:** ^1^ Institute of Infection and Immunity of Huaihe Hospital, Henan University, Kaifeng, China; ^2^ School of Physical Education, Henan University, Kaifeng, China; ^3^ College of Biology Science and Engineering, Hebei University of Economics and Business, Shijiazhuang, Hebei, China

**Keywords:** *Inonotus sanghuang*, polyphenols, inflammation, obesity, NF-κB signaling, MAPK signaling

In the original article, there was a mistake in the total STAT3 expression of western blotting data in **Figure 7B** as published. There was an unintentional error in the **Figure 7B**. The corrected **Figure 7** appears below. The authors apologize for this error and state that this does not change the scientific conclusions of the article in any way. The original article has been updated.

**Figure 7:**
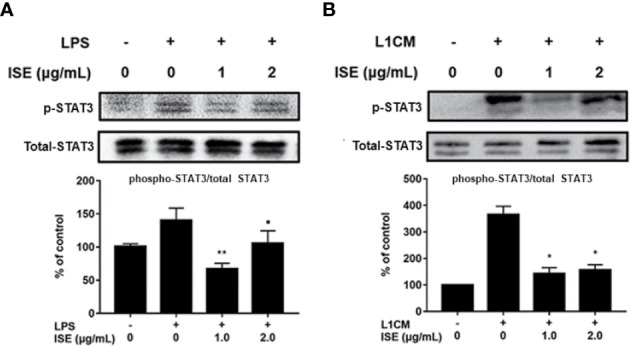
Effects of ISE on STAT3 signal. RAW264.7 macrophages were pretreated with different concentrations of ISE for 1h and then stimulated by 1.0μg/mL LPS or 3T3-L1CM for 30min. Total cell lysates were extracted, and then western blotting using specific antibodies was used to determine the expression of p-STAT3 and STAT3 in LPS **(A)** and 3T3-L1CM (L1CM) stimulation **(B)**, respectively. The value of a control was set at 100%, and the relative value was presented as fold induction to that of the control, which was normalized to total STAT3. Statistical comparisons were made with each vehicle controls. The values are means ± SD, *n* = 3. *P < 0.05, **P < 0.01.

