# Supplementary material for: Inonotus sanghuang Polyphenols Attenuate Inflammatory Response Via Modulating the Crosstalk Between Macrophages and Adipocytes
Source: Front Immunol. 2019 Feb 26;10:286. doi: 10.3389/fimmu.2019.00286 (PMC6399398; doi:10.3389/fimmu.2019.00286)
Supplement: Supplementary file 1 [file Table_1.DOCX]

**
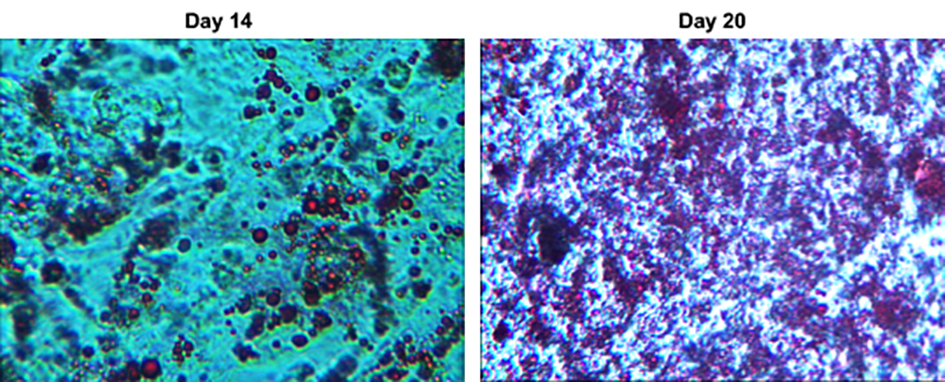
**

**Supplementary Figure 1.** Hypertrophied 3T3-L1 adipocytes were observed by oil red O staining day 14 and 20 after 3T3-L1 adipocytes were differentiated as described in “Materials and Methods”.
